# Use of Preoperative 3D Virtual Planning and 3D-Printed Patient-Specific Guides to Facilitate a Single-Stage Cranial Closing Wedge Ostectomy and Tibial Plateau Leveling Osteotomy Procedure to Address Proximal Tibial Deformity, an Excessive Tibial Plateau Angle, and Cranial Cruciate Ligament Insufficiency in a Dog

**DOI:** 10.1155/2023/3368794

**Published:** 2023-11-23

**Authors:** Christina C. De Armond, Daniel D. Lewis, Sarah Townsend

**Affiliations:** Department of Small Animal Clinical Sciences, College of Veterinary Medicine, University of Florida, Gainesville, FL 32610, USA

## Abstract

A 9-month-old mixed-breed dog was presented for bilateral proximal tibial deformity resulting in an excessive tibial plateau angle and cranial cruciate ligament insufficiency. Initial surgical management of the right pelvic limb was done by performing a cranial closing wedge ostectomy. Inadequate leveling of the plateau resulted in a postliminal meniscal tear which was addressed during a revision tibial plateau leveling osteotomy. The left pelvic limb was managed in a single-session surgery using three-dimensional (3D) virtual surgical planning and custom 3D-printed surgical guides to perform a combined cranial closing wedge ostectomy and tibial plateau leveling osteotomy. Postoperative 3D analysis of the left tibia revealed the accuracy of the surgical result within 2° of the virtual surgical plan. The dog developed a transient grade II/IV left medial patellar luxation following surgery but ultimately attained a full functional recovery and was actively engaged in competitive agility work 46 months following surgery on the left pelvic limb.

## 1. Introduction

Excessive tibial plateau angle (eTPA) has been defined as a tibial plateau angle (TPA) greater than 34° [1–5]. Addressing CCL insufficiency in dogs with an eTPA presents a challenge to veterinary surgeons. Tibial plateau leveling osteotomy (TPLO) is commonly performed to manage CCL disease, but obtaining adequate rotation of the plateau segment can be difficult in dogs with an eTPA. Rotation of the plateau segment beyond the apex of the tibial tuberosity, which is often required in dogs with an eTPA, increases the risk of tibial tuberosity fracture [3, 6, 7]. Outcomes in dogs with eTPA, treated by various surgical techniques, are associated with a higher complication rate, necessitating additional surgery nearly 8 times more frequently than in dogs with a normal TPA [3, 4].

Combining a cranial closing wedge ostectomy (CCWO) with a TPLO has been advocated to address CCL insufficiency in dogs with an eTPA to effectively level the tibial plateau [3]. The combined CCWO/TPLO technique has resulted in favorable long-term outcomes but carries an overall complication rate of 78%, with 33% of complications being classified as major [3]. The reported complications include implant loosening or failure and inconsistency in obtaining an appropriate postoperative TPA [3].

The use of three-dimensional (3D) virtual surgical planning and the use of 3D-printed custom surgical guides have gained interest in veterinary orthopedics [8–14]. Custom guide design is typically reserved for complex procedures, such as deformity correction and vertebral stabilization [8–14]. The proposed benefits of this technology include reduced fluoroscopy use and improved surgical precision [11, 14–16]. This report describes the use of preoperative 3D virtual surgical planning and 3D-printed custom surgical guides to perform a single-stage combined CCWO/TPLO procedure to address an eTPA of 78° in a mixed-breed dog with CCL insufficiency.

## 2. Case Report

**Figure 1:**
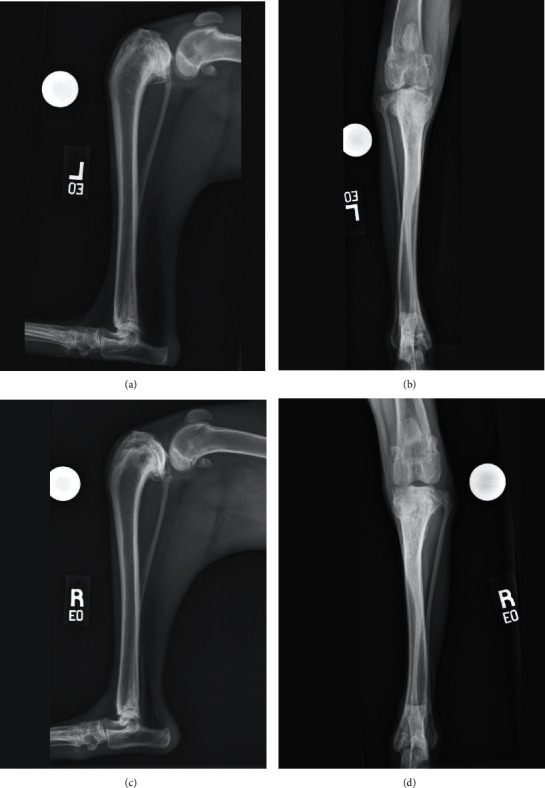
(a, b, c, d) Radiographs of both crura at the time of presentation. There is a profound deformity of the proximal tibia resulting in an excessive tibial plateau angle, which was measured to be approximately 70°. There is cranial displacement of the proximal tibia, consistent with cranial cruciate ligament insufficiency.

A 9-month-old male neutered 24.5 kg mixed-breed dog presented to the University of Florida Small Animal Hospital for the management of bilateral CCL insufficiency and eTPA characterized by proximal tibial and fibular deformity. Lameness was first noted at 4 months of age but had recently become more severe. On presentation, the dog had profound bilateral pelvic limb lameness characterized by a crouched pelvic limb posture and a shortened, shuffling stride. Both stifles were circumferentially swollen, with firm thickening over the medial aspect of the stifle. Moderate stifle effusion was present bilaterally, and neither stifle could be fully extended. A moderate pain response was elicited while assessing the stifle range of motion. Cranial drawer and cranial tibial thrust were difficult to elicit in both stifles. A grade I/IV medial luxation of the left patella was also noted. Orthogonal view radiographs of both crura and distal femora were obtained which revealed bilateral proximal tibial deformity characterized by excessive proximal procurvatum, resulting in an eTPA of approximately 70°, caudoproximal displacement of the tibial tuberosity, cranial subluxation of the tibia, slight patella alta, and mild secondary degenerative changes (Figures 1(a)–1(b)). The deformity was presumed to be developmental, most likely secondary to eccentric premature closure of the caudal proximal tibial physes.

**Figure 2:**
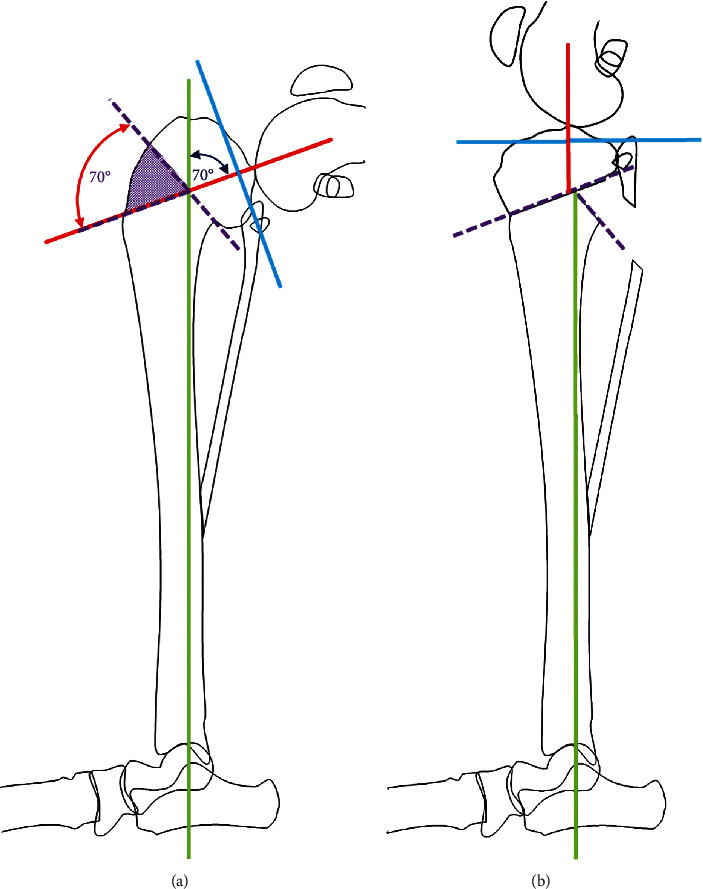
(a, b) Schematic representation of preoperative surgical planning for CCWO of the right tibia based on the preoperative mediolateral radiograph.

**Figure 3:**
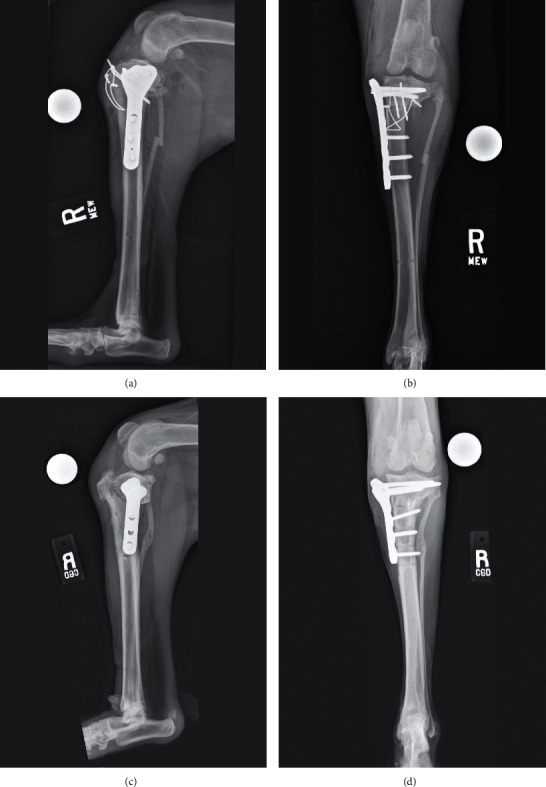
(a, b) Initial postoperative radiographs of the right crus after a cranial closing wedge ostectomy was performed. Note that the femoral condyles are positioned centrally over the tibial plateau, but the postoperative TPA was 20° with some minor valgus of the proximal tibia and external torsion. (c, d) Postoperative radiographs obtained following a revision procedure in which a tibial plateau leveling osteotomy was performed. The final TPA was 4° with improvement in the valgus and torsional deformity induced by the initial surgery.

The initial plan was to perform surgery on the right tibia first and reduce the eTPA using a CCWO (Figures 2(a) and 2(b)) [17]. A medial parapatellar stifle arthrotomy revealed a complete rupture of the CCL and intact menisci. A 1 cm segmental proximal fibular ostectomy was performed via a limited lateral approach to the fibula. Via an approach to the proximomedial tibia, a 70° CCWO was performed in the proximal tibia. Reduction of the CCWO was not possible due to caudal soft tissue tension, so an additional 1.5 cm segment of bone had to be excised from the proximal margin of the distal tibial segment to mitigate soft tissue tension. The ostectomy was stabilized using a medially positioned 3.5 mm broad locking CBLO plate (Veterinary Orthopedic Implants, St. Augustine, Florida, USA), an interfragmentary 1.6 mm Kirschner wire, and a cranially positioned tension band wire. The stifle was assessed for stability; cranial drawer could, but cranial tibial thrust could not be elicited. Postoperative radiographs revealed appropriately positioned osteotomies and implants, with the femoral condyles positioned centrally over the tibial plateau (Figures 3(a) and 3(b)). There was some minor proximal tibial valgus and external tibial torsion induced compared to the preoperative limb. The resultant postoperative TPA was 20°. Initial surgical recovery was uneventful, and the dog was fully weight-bearing but still maintained a crouched pelvic limb posture while ambulating when reevaluated 1 month following surgery. Examination at that time revealed an improved range of motion in the right stifle; cranial tibial drawer, but not cranial tibial thrust, could be elicited under sedation. Radiographs revealed early healing of the osteotomies and patellar tendon thickening, suspected to be a transient postoperative finding secondary to leveling osteotomy. No implant-related complications were noted.

The dog became acutely lame on the right pelvic limb 85 days after surgery, and the owners noted an audible click while the dog was ambulating. The dog returned to the UF SAH, and an orthopedic examination revealed a shifting pelvic limb lameness with bilateral stifle effusion and thickening. Cranial drawer, cranial tibial thrust, and a presumptive meniscal click could be elicited in the right stifle. A postliminary meniscal tear was suspected. The dog was taken to surgery, and a medial parapatellar arthrotomy revealed a bucket handle tear of the caudal horn of the medial meniscus which was excised. The CBLO plate and screws as well as the tension band fixation were removed. A TPLO was performed and stabilized using a 3.5 mm broad locking TPLO plate (DePuy Synthes, Raynham, MA, USA). Postoperative radiographs revealed appropriately positioned implants and a TPA of 4° (Figures 3(c) and 3(d)). Recovery from the second surgery was uneventful, and limb function improved. On examination 1 month postoperatively, the dog's right pelvic limb lameness had improved, but the crouched posture in both pelvic limbs persisted. The right stifle was comfortable on palpation, cranial tibial thrust could not be elicited, and effusion was decreased. Radiographs obtained at that time revealed early healing of the TPLO without apparent complications.

The owners wanted to pursue surgical intervention of the left pelvic limb. A single session of combined CCWO/TPLO was recommended, aided by 3D virtual surgical planning and 3D-printed custom surgical guides to execute efficient resolution of the tibial conformational abnormalities and stifle instability. Computed tomography (CT) (160 Slice Toshiba Aquillion CT Scanner, Cannon Medical Systems, Tustin, CA, USA) imaging of both pelvic limbs was performed (slice thickness of 0.5 mm and slice overlap of 0.3 mm). Bone volume DICOMs were imported into 3D medical imaging software (Materialise Mimics, Materialise Medical Software Suite, Materialise, Leuven, Belgium) to render a 3D virtual model stereolithography file (.stl) of the left pelvic limb. The .stl file was then imported into 3D modeling software (Materialise 3Matic, Materialise Medical Software Suite, Materialise, Leuven, Belgium) for virtual surgical planning and custom guide design.

The TPA of the left tibia was measured at 78° using traditional tibial plateau landmarks [5] from the virtually segmented bone model (Materialise 3Matic, Materialise Medical Software Suite, Materialise, Leuven, Belgium) which was 8° greater than the TPA measured on the prior radiographs. As part of the virtual surgical plan, a 48° CCWO was performed, which was initiated 4 cm distal to the tibial tuberosity. To shorten the tibia, an additional 2.4 cm segmental ostectomy was incorporated at the distal margin of the CCWO. The apposition of the tibial segment margins reduced the TPA to 24°. To further level the plateau, a virtual TPLO was performed using a 27 mm radial osteotomy rotated 8.8 mm, yielding a final TPA of 5°. The distal tibial segment was also internally rotated 3° to align the tibial tuberosity with metatarsal bones III and IV [18].

**Figure 4:**
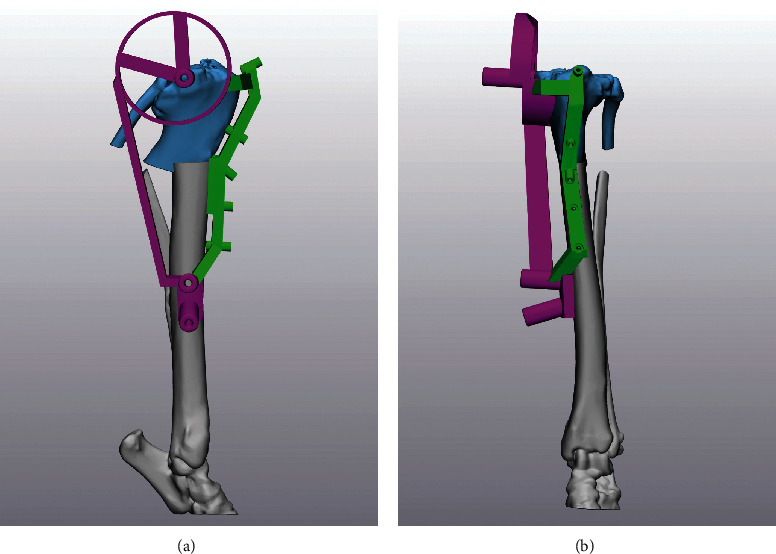
(a, b) Orthogonal images of the virtually corrected tibia with the hybrid TPLO/reduction guide (magenta) positioned on the medial surface of the tibia and the adjoining cranial reduction guide (green) positioned on the cranial tibia.

**Figure 5:**
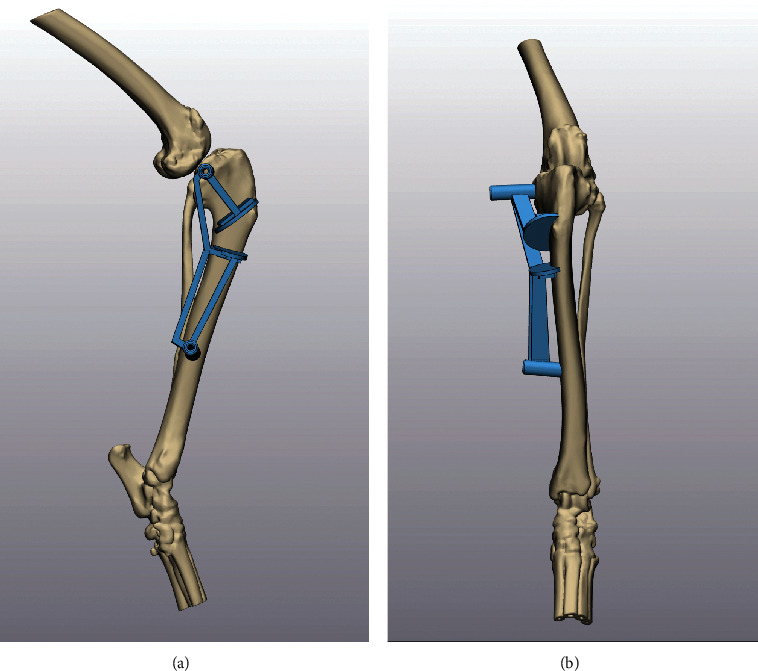
(a, b) Orthogonal images of the uncorrected virtual tibia with the cranial closing wedge/segmental tibial excision osteotomy guide (blue) positioned over the medial surface of the proximal tibia.

Based on the virtual surgical plan, a series of custom guides were designed [14]. First, a cranial reduction guide was designed to maintain the reduction and alignment of the osteotomies during the placement of a medial plate. This guide consisted of material that conformed to the topography of the cranial surface of the virtually corrected tibia with five drill guide towers designed to accommodate 1.6 mm Kirschner wires inserted from cranial-to-caudal at converging and diverging angles (Figures 4(a) and 4(b)). Next, an adjoining hybrid TPLO/reduction guide was designed to be positioned over the medial surface of the tibia following CCWO. This guide articulated with the cranial reduction guide and consisted of three drill guide towers for 3.2 mm pin (Duraface® Fixation Half-pins, IMEX Veterinary, Longview, Texas, USA) placement in the proximal tibia and mid-distal tibial diaphysis, similar to traditional TPLO jig pin placement [6], with an additional converging distal pin to compress the guide onto the tibial cortex. The proximal portion of the guide was designed with a 3 mm offset to accommodate the medial buttress. The amount of offset was an estimation—if an additional buttress was noted during surgery, it would have been dissected so that the guide position was not altered by soft tissues. The proximal aspect of this guide also incorporated a cylindrical osteotomy guide designed to accommodate a 27 mm TPLO saw blade (Colibri II, DePuy Synthes, Warsaw, Indiana, USA) (Figures 4(a) and 4(b)). Once the reduction guides were designed, the virtually corrected tibial segments were superimposed over the intact tibia, and the initial CCWO, including the excision of a 2.4 cm segment of bone from the proximal aspect of the distal tibial segment, was planned. A medially positioned osteotomy guide was designed using the centrally located virtually corrected tibial segments to dictate the locations of the proximal and distal tibial osteotomies [14] (Figures 5(a) and 5(b)).

Models of preoperative and virtually corrected bones and guides were printed using biocompatible plastics (PC-ISO and ABS-M30i, Stratasys, Eden Prairie, Minnesota, USA) on a fused deposition modeling 3D printer (Fortus 450mc, Stratasys, Eden Prairie, Minnesota, USA). Rehearsal surgery was performed using printed guides and bones prior to surgery. A 3.5 mm conical coupling locking plate (Fixin, Intrauma S.p.A., Rivoli, Italy) was precontoured to the cranial surface of the tibia model, bridging the CCWO. All guides were sterilized using ethylene oxide gas, per the manufacturer's guidelines (PC-ISO and ABS-M30i, Stratasys, Eden Prairie, Minnesota, USA).

### 2.1. Surgery

**Figure 6:**
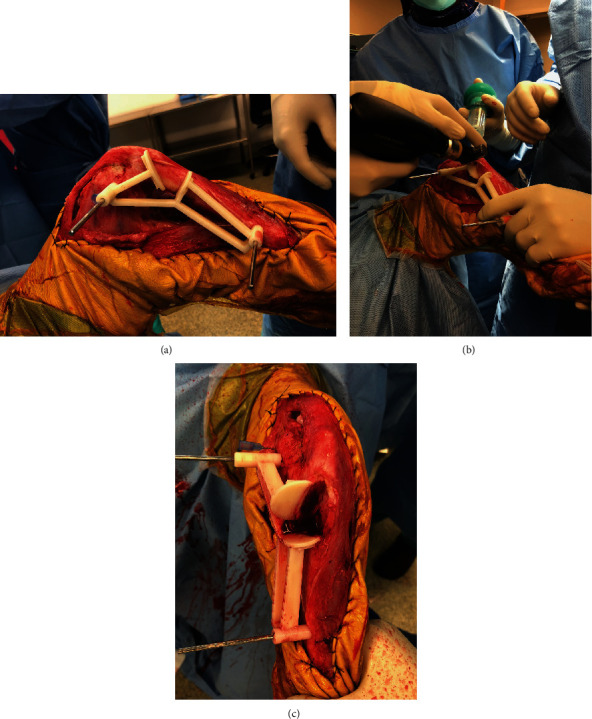
Intraoperative use of the 3D-printed osteotomy guide. (a) The cranial closing wedge ostectomy guide was secured to the medial surface of the tibia using 3.2 mm threaded pins. (b) The cutting shelves were used to precisely incise the tibia with a sagittal saw. (c) After completing the osteotomies, the free central tibial segment was removed. The guide was removed, but the pins securing the guide were left in place. The TPLO/reduction guide subsequently was placed over these pins to align and reduce the tibia.

**Figure 7:**
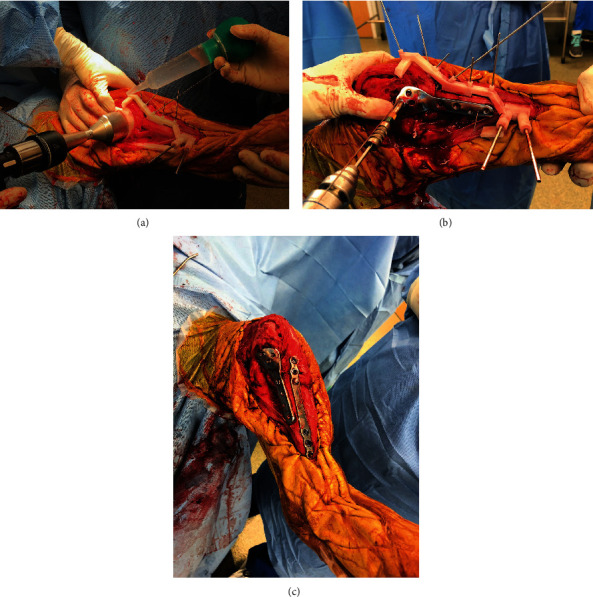
Intraoperative use of the TPLO/reduction and adjoining cranial reduction guides. (a) The TPLO/reduction guide was secured over the previously implanted parallel pins, which initially secured the cranial closing wedge osteotomy guide, to reduce and align the proximal and distal tibial segments. The cranial reduction guide was placed, and the proximal portion of the TPLO/reduction guide was used to direct the TPLO saw. (b) After the rotation of the plateau segment, an interfragmentary Kirschner wire was placed through the most proximal guide tower of the cranial reduction guide to maintain the rotation. The medially positioned TPLO/reduction guide was removed. The cranial reduction guide maintained the alignment of the tibial as the CBLO plate was applied. (c) After removing the cranial reduction guide, a precontoured conical coupling plate was secured to the cranial tibia to provide supplemental fixation.

**Figure 8:**
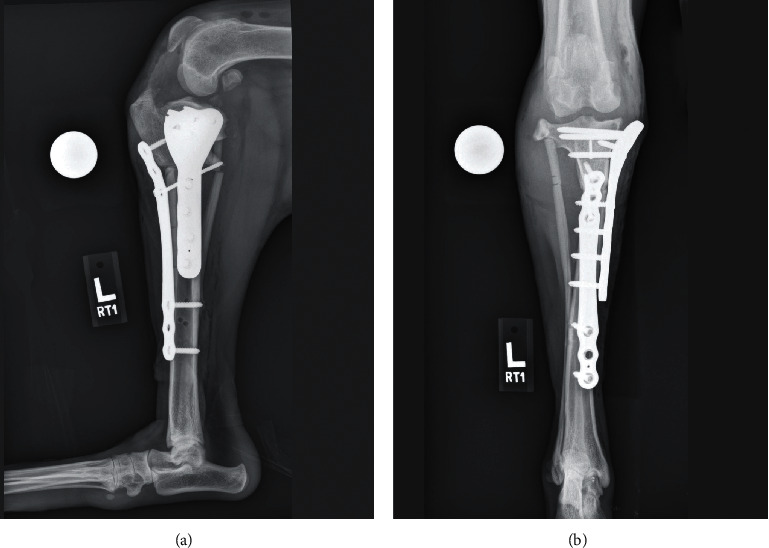
(a, b) Initial postoperative radiographs of the left crus. The postoperative tibial plateau angle was estimated to be 8° based on these radiographs (later confirmed to be 7° on a subsequent CT), and the femoral condyles are positioned centrally on the tibial plateau.

The dog was placed in dorsal recumbency for surgery. A medial approach to the stifle was performed, and the approach was extended distally to the level of the mid-tibial diaphysis. A limited medial parapatellar arthrotomy was performed which confirmed a complete tear of the CCL and intact menisci. The CCWO osteotomy guide was secured to the medial surface of the tibia using two 3.2 mm threaded pins (Duraface® Fixation Half-pins, IMEX Veterinary, Longview, Texas, USA) (Figure 6). The appropriate guide location was subjectively easily identified. A sagittal saw (Colibri II, DePuy Synthes, Warsaw, Indiana, USA) was used to initiate the CCWO osteotomies using the custom guide cutting shelves (Figures 6(b) and 6(c)). Prior to completing the tibial excision, a limited lateral approach was made to the proximal fibula, and three subperiosteal and transverse osteotomies were made adjacent to the location of the CCWO. The proximal free segment of the ostectomized fibula was removed. Excision of the tibial segment was completed, and the cancellous bone within the segment was harvested to be used as a bone graft. The custom osteotomy guide was removed, and the TPLO/reduction guide was slid over the previously placed proximal and distal pins, aligning the proximal and distal tibial segments. The guide was secured to the tibia by placing an additional distal converging pin. The cranial reduction guide was applied and secured using four 1.6 mm Kirschner wires placed for temporary stabilization. Using the custom TPLO/reduction guide, a 27 mm TPLO saw blade [5] was used to create a radial osteotomy in the proximal tibia, and the plateau segment was rotated 8.8 mm (Figure 7). The rotation was maintained by placing a final proximal interfragmentary Kirschner wire through the cranial reduction guide. Cranial tibial thrust could not be elicited. The TPLO/reduction guide was removed, and the osteotomies were stabilized using a 3.5 mm broad-locking CBLO (Veterinary Orthopedic Implants, St. Augustine, Florida, USA) plate applied to the medial surface of the proximal tibia (Figure 7(b)). The Kirschner wires and cranial reduction guide were removed. The precontoured six-hole 3.5 mm conical coupling locking plate (Fixin, Intrauma S.p.A., Rivoli, Italy) was applied to the cranial tibia (Figure 7(c)). The cancellous bone harvested from the excised tibial segment was packed into the tibial ostectomy site. Postoperative radiographs revealed appropriate alignment of the tibia and fibula, placement of the implants, and positioning of the femoral condyles relative to the tibial plateau. The TPA was estimated to be 8° but could not be definitely measured as the plateau was partially obscured by the CBLO plate (Figures 8(a) and 8(b)).

### 2.2. Outcome

The dog recovered uneventfully from surgery. The dog was reexamined at the UF SAH 1 month following surgery and had mild weight-bearing left pelvic limb lameness but no longer had a crouched pelvic limb posture. Both stifles had a normal range of motion. Cranial drawer, but not tibial thrust, was elicited in both stifles. Periarticular thickening and mild effusion were noted in the left stifle, as was a grade II/IV medial patellar luxation. Radiographs showed evidence of early healing of the osteotomies without apparent implant-associated complications. The owners were advised to initiate physical therapy aimed at strengthening the quadriceps muscles. The lameness and the patellar luxation had resolved when the dog was reexamined 3 months postoperatively. Radiographs performed at this time showed that the osteotomies had obtained osseous union without complications. A CT of the pelvic limbs was performed to determine the accuracy of the surgical execution of the virtual plan. DICOMs were processed in the same manner as the preoperative CT. A 3D rendering of the postoperative limb revealed an increase of 1° varus in the frontal plane when compared to the virtual plan. The measured postoperative TPA was 7°, which was 2° greater than the virtual plan.

**Figure 9:**
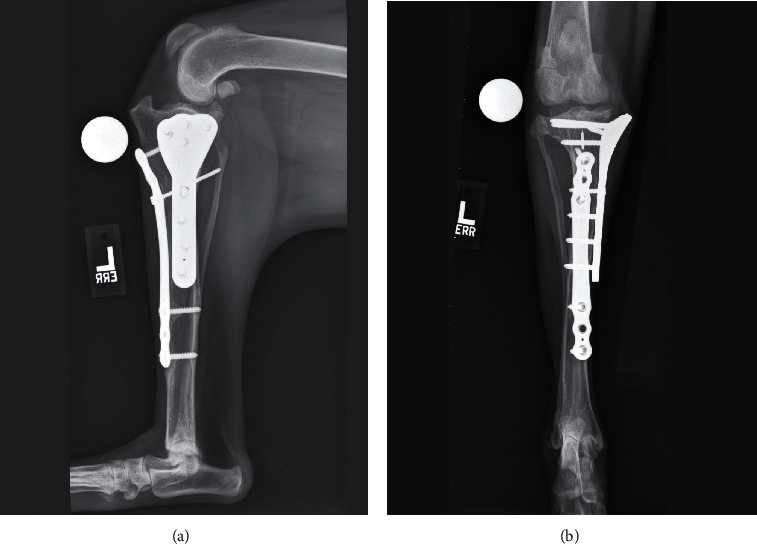
(a, b) Long-term follow-up radiographs of the left crus were obtained 46 months after surgery. There has been no loss of tibial alignment. The tibial and fibular osteotomies have healed and remodeled. The femoral condyles are positioned centrally on the tibial plateau, and there has been nominal progression of the degenerative joint disease affecting the stifle.

The owners were solicited to return the dog to the UF SAH for a long-term follow-up evaluation 1,386 days following surgery. The owner reported that the dog's pelvic limb function was normal, and the dog was actively engaged in agility training. On examination, the dog ambulated without apparent lameness. Cranial drawer could be elicited in both stifles, but neither stifle had cranial tibial thrust. Neither patella could be luxated. There was mild periarticular thickening of both stifles, but no palpable effusion. Force plate analysis obtained from a trot (average velocity 1.83 ± 0.10 m/sec) revealed a <1% difference in peak vertical force between the right (69.52 + 3.97100 × *N*/*N*) and left (70.15 + 0.35100 × *N*/*N*) pelvic limbs. Measurements of stifle extension (right stifle: 153°; left stifle: 155°) and flexion (right stifle: 30°; left stifle: 30°) were functional [19], and thigh circumference was similar (right thigh: 27.8 cm; left thigh: 28.4 cm) between limbs. Radiographs of both crus were obtained which revealed continued remodeling of the healed tibia and fibula; no complications were noted in association with the implants; and a nominal progression of osteoarthritic abnormalities, slightly more advanced in the right stifle than the left stifle, as noted in previous radiographic studies. The previously noted thickening of the right patellar tendon had resolved, although there was mineralization of the distal portion of the tendon (Figures 9(a) and 9(b)).

## 3. Discussion

This case report illustrates the utility of using 3D virtual surgical planning and 3D-printed custom guides for the management of severe proximal tibial deformity characterized by an excessively steep TPA. The initial postoperative and long-term functional outcomes were favorable, and guide use was associated with accurate execution of the complex virtual surgical plan during a single-session surgical procedure.

Correction of the proximal tibial deformity associated with eTPA is technically challenging, with a reported complication rate of 40% [4, 5, 7]. Complications encountered when correcting eTPA include patellar tendon thickening, tibial tuberosity and fibular fracture, implant failure, and an increase in the postoperative TPA during the convalescent period as a result of plateau segment rock back [4]. Adequate leveling of eTPA using a TPLO alone has been discouraged due to the risk of tibial tuberosity fracture that results from the loss of buttress support provided by the tibial plateau segment [3, 6, 7]. Correction of eTPA with CCWO alone is associated with changes in femoropatellar mechanics and the development of patella baja due to shortening of the tibia, though the clinical significance of this is unknown [20]. Additionally, CCWO is associated with variable leveling despite careful procedure planning [21], as occurred in the initial surgery on the right pelvic limb in the dog reported here. We suspect that the undercorrection of the eTPA during that initial surgery was in part due to the erroneous characterization of the TPA on the preoperative radiographs. Both proximal tibiae were markedly deformed, which impeded accurate measuring of the TPA. We initially measured the left TPA at 70° on radiographs but were able to more accurately define the TPA at 78° on the subsequent preoperative CT. We performed a 70° CCWO which potentially could have reduced the TPA to 0° because we have had consistent problems with postoperative TPAs being too high following CCWOs in dogs with eTPAs.

Correction of eTPA to ≤14° has been advocated to reduce the risk of postoperative complications in dogs with eTPA [4], but undercorrection of the TPA can result in residual cranial tibial thrust, thereby increasing the risk of developing postliminary meniscal pathology as occurred in the right stifle of the dog in this report [6, 22]. Additionally, undercorrection of an eTPA also reportedly results in diminished client satisfaction [4]. For these reasons, combining a CCWO and TPLO has been advocated to address dogs with eTPA, but this procedure is associated with variability in plateau leveling and a high complication rate, with one-third of dogs requiring additional surgery [3]. We were able to obtain precise plateau leveling in a single procedure by employing virtual surgical planning and custom 3D-printed guides, thus eliminating cranial tibial thrust and preserving the menisci. The dog's subclinical low-grade medial patellar luxation noted during the 1-month postoperative evaluation was transient and resolved without additional surgery.

Performing a large wedge ostectomy of the proximal tibia is associated with strain on muscular and neurovascular structures, particularly the peroneal nerve unless concurrent crural shortening is performed [23]. Additional tibial shortening was necessary to overcome the soft tissue constrains which inhibited the closure of the CCWO during surgery in the right pelvic limb of the dog in this report, but performing a freehand segmental ostectomy potentiates the possibility of inducting iatrogenic frontal and sagittal plane deformities, as occurred in the right stifle of the dog in this case report [24, 25]. A 2.4 cm segment of the tibia was also removed adjacent to the distal margin of the CCWO in the virtual surgical plan for the left pelvic limb to reduce soft tissue tension in a precise manner. The result of using guides to incorporate a shortening component to the CCWO in the left pelvic limb proved to be accurate, as the proximal and distal tibial segments reduced readily with minimal soft tissue tension and without inducing deformity.

Accurate execution of the virtual surgical plan in the operating room relies on the correct positioning of the 3D-printed guides [26, 27]. Guides dictate the location and trajectory of the surgical drill and saw, and inaccuracies can result in a number of complications including inducing iatrogenic deformity [26, 27]. In our case, accurate guide placement was subjectively easily accomplished and is reflected in the minor discrepancies found when comparing the postoperative CT images to the virtual surgical plan. Effective guide application is ascribed to specific details involved in designing the guides. In our experience, designing custom surgical guides is predicated on incorporating sufficient unique osseous topography to facilitate accurate guide placement but must be balanced to allow sufficient access to perform surgical tasks. The guides designed in this case had relatively small areas of osseous contact but utilized specific and prominent osseous landmarks to reduce the potential for improper guide placement. Additionally, models of both the pre- and postcorrection tibia were sterilized and used for intraoperative reference, further increasing the probability of proper guide placement.

The disadvantages of 3D virtual surgical planning and 3D-printed custom surgical guides include the additional cost and time, as well as the required training [28]. These limitations have confined the use of this technological advancement to more complicated and potentially less time-sensitive surgical challenges, but the increasing availability of 3D printers and open-source software may result in 3D technology becoming more commonplace in the operating room [29–34].

The use of 3D virtual surgery and 3D-printed custom surgical guides facilitated the accurate execution of a single-stage surgical procedure to address this dog's complex tibial deformity. This is only a single case report with long-term follow-up data; however, the authors feel that virtual surgical planning and surgical guides were useful for this challenging case. Future controlled prospective case studies would be beneficial to determine the potential superiority of this technique over traditional planning and surgical modalities in managing dogs with CCL insufficiency associated with eTPA.

## Data Availability

The data is available on request.
